# Temperature-Adaptive Carrier Regulation and Enhanced Thermoelectric Performance in n-Type PbTe via Deep-Shallow Co-Doping

**DOI:** 10.3390/ma19132832

**Published:** 2026-07-02

**Authors:** Aihua Song, Peng Zhao, Binhao Wang, Dan Wang, Chen Chen, Tao Shen, Hang Li, Bo Xu, Yongjun Tian

**Affiliations:** 1 Center for High Pressure Science (CHiPS), State Key Laboratory of Metastable Materials Science and Technology, Yanshan University, Qinhuangdao 066004, China; sah0316@163.com (A.S.); wangdan120805@126.com (D.W.); c-chen@tsinghua.edu.cn (C.C.); shentao4019@126.com (T.S.); hangli@stumail.ysu.edu.cn (H.L.); fhcl@ysu.edu.cn (Y.T.); 2 State Key Laboratory for High Performance Tools, Zhengzhou Abrasive Grinding Research Institute Co., Ltd., Zhengzhou 450001, China; wbh_ysu@126.com

**Keywords:** thermoelectric materials, n-type PbTe, Ga/I co-doping, carrier concentration, power factor

## Abstract

Optimizing the carrier concentration across the entire operating temperature range is crucial for maximizing the power factor in n-type PbTe. However, conventional shallow donors produce a nearly temperature-invariant electron concentration, leading to an increasingly large deviation from the optimal carrier concentration at elevated temperatures. Herein, we implement a dynamic deep-shallow co-doping strategy by combining iodine (a shallow donor) with gallium (a deep-level donor) in PbTe. The Ga-related deep impurity states thermally ionize at elevated temperatures, providing additional electrons and driving the Hall carrier concentration above ~563 K toward its temperature-dependent optimum. Concurrently, our optimized synthesis preserves a high carrier mobility, which synergistically sustains a remarkable peak power factor of 30 μW·cm^−1^·K^−2^ for the optimal composition, Ga_0.02_Pb_0.98_Te_0.996_I_0.004_. Combined with a strongly suppressed lattice thermal conductivity, this results in a maximum figure of merit (*ZT*) of 1.41 at 803 K and an average *ZT* of 1.00 within 400–773 K for Ga_0.02_Pb_0.97_Te_0.996_I_0.004_—a 25% improvement over the I-only doped baseline. These findings establish deep-shallow co-doping as a robust and broadly applicable carrier-engineering paradigm for thermoelectric optimization.

## 1. Introduction

Thermoelectric materials enable the direct conversion of heat into electricity and vice versa, offering a promising avenue for waste-heat recovery and solid-state refrigeration [1,2,3]. The energy-conversion efficiency of a thermoelectric device is governed by the dimensionless figure of merit, *ZT* = *S*^2^*σT*/*κ*, where *S*, *σ*, *T*, and *κ* represent the Seebeck coefficient, electrical conductivity, absolute temperature, and total thermal conductivity, respectively. The term *S*^2^*σ* is defined as the power factor (*PF*), which characterizes the electronic transport properties. Maximizing *ZT* fundamentally requires simultaneously attaining a high *PF* and a low *κ*; however, these parameters are strongly coupled through the carrier concentration [4,5,6]. Therefore, precise carrier-concentration optimization remains one of the most effective approaches to decouple and balance electrical and thermal performance [7,8,9,10].

Lead telluride (PbTe) is a benchmark mid-temperature (400–800 K) thermoelectric material, distinguished by its high *S* and *σ*, as well as intrinsically low *κ* [11,12,13]. Nevertheless, the thermoelectric performance of n-type PbTe historically lags behind that of its p-type counterpart. This disparity primarily stems from the larger energy offset between the light and heavy conduction bands (Δ*E*_c_ ≈ 0.45 eV) [14,15,16,17], which significantly restricts the development of PbTe-based thermoelectric devices. Current optimization strategies for n-type PbTe predominantly focus on enhancing the *PF* (e.g., via band-structure flattening [18,19], resonant levels [20,21] and Anderson localization [22]) and reducing *κ* through alloying and microstructure engineering [23,24,25,26,27]. Underlying all these approaches, the Hall carrier concentration (*n*_H_) plays a central role, as it dictates the delicate balance between electrical and thermal transport, and thus must be synergistically tuned to achieve a high thermoelectric performance.

Crucially, the optimum carrier concentration scales as *n*_opt_~(m*T)^1.5^ and therefore increases monotonically with temperature, where m* is the density-of-states effective mass [5,28]. Conventional doping with shallow donors, such as I, Bi, and Sb [21,29,30,31], typically yields a nearly constant *n*_H_, resulting in suboptimal transport at elevated temperatures. To optimize thermoelectric performance over a wide temperature range, deep defect states (e.g., introduced by Ga and In) have been utilized to achieve a temperature-dependent carrier concentration, allowing *n*_H_ to better track the optimum carrier concentration curve [32,33,34,35]. Building on this concept, Zong et al. recently reported Ga/I co-doped PbTe, demonstrating that I improves the solubility of Ga while Ga regulates the carrier concentration via deep defect states [36]. However, conventional synthesis routes for these systems often struggle to balance competing transport properties. Specifically, unoptimized processing and excessive carrier concentrations frequently induce severe carrier scattering, restricting carrier mobility and yielding only moderate thermoelectric performance [36]. Consequently, achieving a synergistic balance between dynamic carrier generation and mobility retention remains a significant challenge.

To address this limitation, the primary contribution of this study is the optimization of the carrier-concentration window and the preservation of high carrier mobility through a carefully modified synthesis protocol. In this work, we systematically investigate a series of Ga/I co-doped n-type PbTe samples synthesized via an optimized melting, annealing and spark plasma sintering (SPS) route. In this synergistic system, iodine provides an appropriate baseline electron concentration, while the Ga-induced deep defect states progressively ionize at elevated temperatures, enabling dynamic, temperature-adaptive carrier concentration optimization. By maintaining the carrier concentration within a favorable regime without incurring the severe mobility penalties characteristic of heavily doped systems, Ga incorporation significantly enhancing carrier mobility, while intensified point-defect scattering markedly suppresses the lattice thermal conductivity (*κ*_L_). Driven by these synergistic effects, we achieved a peak *ZT* of 1.41 and a high average *ZT* of 1.00 with the temperature range of 400–773 K, demonstrating the viability of this co-doping strategy for enhanced overall thermoelectric performance of n-type PbTe.

## 2. Methods

### 2.1. Experimental Section

I-doped and Ga/I co-doped n-type PbTe polycrystalline bulks were synthesized via a combined melting, annealing, and spark plasma sintering (SPS; Sinter Land Inc., Niigata, Japan) process. High-purity elemental precursors—Pb (granules, 99.99%), Te (granules, 99.99%), PbI_2_ (powder, 99.99%), and Ga (ingot, 99.99%)—were stoichiometrically weighed according to the nominal compositions PbTe_1−*x*_I*_x_* (*x* = 0.002, 0.003, 0.004, 0.005) and Ga*_y_*Pb_1−*y*_Te_0.996_I_0.004_ (*y* = 0.01, 0.02, 0.03, 0.04). The mixtures were loaded into carbon-coated quartz ampoules, evacuated to a base pressure of ~10^−4^ Torr and flame-sealed. The ampoules were then heated to 1273 K at a rate of 240 K·h^−1^, held at this temperature for 6 h to ensure complete melting, and subsequently cooled at 240 K·h^−1^ to 873 K. At this stage, the ingots were annealed for 50 h to promote compositional homogeneity before being naturally cooled to room temperature. The resulting ingots were mechanically cleaned, crushed, and ground into fine powders. Finally, these powders were consolidated into dense bulk pellets via SPS at 823 K for 5 min under an axial pressure of 50 MPa in a vacuum environment. These specific sintering parameters were optimized to achieve near-theoretical density (>97%) while effectively suppressing Te volatilization, compositional deviation, and excessive grain growth—factors that are critical for preserving high carrier mobility in the PbTe matrix. To prevent oxidation, all weighing and grinding procedures were conducted in an Ar-filled glovebox.

The crystallographic phase purity of the sintered samples was verified with X-ray diffraction (XRD; D/Max-2500/PC X-ray diffractometer, Rigaku Corporation, Tokyo, Japan). Microstructural morphology and elemental distribution were characterized using scanning electron microscopy (SEM; Thermo Fisher Helios 5 CX DualBeam, Thermo Fisher Scientific, Waltham, MA, USA) equipped with an energy-dispersive X-ray spectrometer (EDS). For transport properties evaluation, the SPS bulks were cut into specific geometries: rectangular bars (2 × 2 × 8 mm^3^) for electrical measurement, and squared slices (6 × 6 × 1 mm^3^ and 6 × 6 × 0.5 mm^3^) for thermal and Hall measurements, respectively. The temperature-dependent electrical conductivity and Seebeck coefficient were simultaneously measured using a ZEM-3 apparatus (Ulvac Riko/ADVANCE RIKO, Inc., Yokohama, Japan). The total thermal conductivity was determined using the relationship *κ* = *DαC*_p_. Here, the bulk density (*D*) was measured via the Archimedes method as listed in Table S1, and the thermal diffusivity (*α*) was obtained using a laser flash apparatus (Netzsch, LFA-457, NETZSCH-Gerätebau GmbH, Selb, Germany). For *T* > 300 K, the specific heat capacity was estimated based on the empirical relationship for lead chalcogenides, *C*_p_/*k*_B_ per atom = 3.07 + 4.7 × 10^−4^ × (*T* − 300) [14], as detailed in Figure S1. Temperature-dependent Hall coefficients (*R*_H_) were acquired using a Hall-effect measurement system (HMS-8400, Lake Shore Cryotronics, Inc., Westerville, OH, USA). The corresponding Hall carrier concentration (*n*_H_) and Hall mobility (*μ*_H_) were derived using the standard relations *n*_H_ = 1/(*e*·*R*_H_) and *μ*_H_ = *σ*·*R*_H_, respectively, where *e* is the elementary charge.

### 2.2. Theoretical Calculation

To elucidate the impurity-derived electronic states induced by Ga and I doping, first-principles calculations were performed based on density functional theory (DFT) as implemented in the Vienna ab initio simulation package (VASP.5.4.4) [37,38,39]. The exchange-correlation interactions were described using the generalized gradient approximation (GGA) parameterized Perdew–Burke–Ernzerhof (PBE) [40,41]. A plane-wave basis set with a cutoff energy of 400 eV was employed, and the Brillouin zone was sampled using a Γ-centered Monkhorst–Pack *k*-point mesh [42] of 4 × 4 × 4 for both geometry optimization and subsequent density-of-states (DOS) calculations. The initial structural model comprised a 2 × 2 × 2 supercell containing 32 Pb atoms and 32 Te. Following volume optimization of the pristine supercell, defect configurations were constructed by substituting specific lattice sites: I on Te sites (Pb_32_Te_31_I), Ga on Pb sites (GaPb_31_Te_32_), and their co-doped configuration (GaPb_31_Te_31_I). These defect structures were fully relaxed until the maximum Hellmann-Feynman force on each atom was less than 0.01eV Å^−1^. Finally, given the presence of heavy elements, spin–orbit coupling (SOC) effects were explicitly incorporated in all electronic structure calculations to ensure accurate derivation of the band structures and DOS.

## 3. Results and Discussion

**Figure 1:**
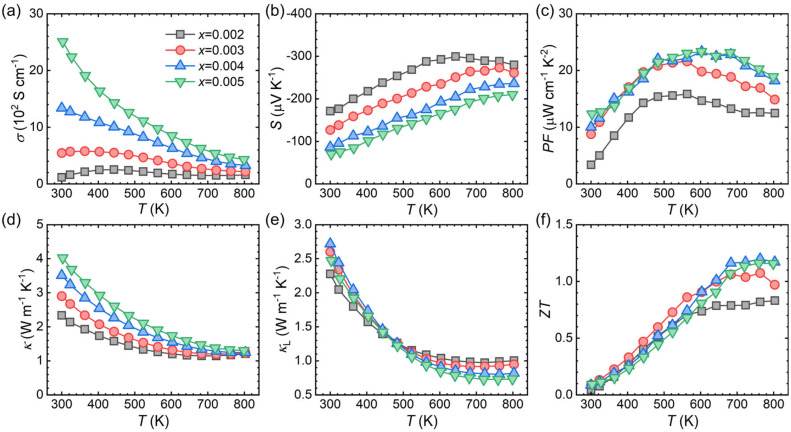
Thermoelectric performance of PbTe_1−*x*_I*_x_* (*x* = 0.002, 0.003, 0.004, 0.005) as a function of temperature: (**a**) electrical conductivity; (**b**) Seebeck coefficient; (**c**) power factor; (**d**) total thermal conductivity; (**e**) lattice thermal conductivity; (**f**) dimensionless figure of merit, *ZT*.

To establish an n-type PbTe matrix with an optimal electron concentration, we first synthesized a series of I-doped samples, PbTe_1−*x*_I*_x_* (*x* = 0.002, 0.003, 0.004, 0.005). The X-ray diffraction (XRD) patterns of these samples are presented in Figure S2, and their thermoelectric properties are summarized in Figure 1. Iodine doping significantly enhances the electrical conductivity, confirming its role as an efficient shallow donor in PbTe; this is consistent with the temperature-dependent Hall carrier concentration data shown in Figure S3. As expected from the carrier-concentration dependence, the Seebeck coefficient decreases with increasing I content (Figure 1b). Consequently, the power factor is substantially enhanced with higher I content across the entire measured temperature range (Figure 1c). Figure 1d shows that the total thermal conductivity increases with increasing I content. The lattice thermal conductivity is estimated using the relationship *κ*_L_ = *κ* − *LσT*, where the Lorenz number (*L*) is estimated according to the empirical model, *L* = (1.5 + exp(−|*S*|/116)) × 10^−8^ V^2^ K^−2^ [43]. At lower temperature (<500 K), *κ*_L_ exhibits comparable values across compositions; however, at elevated temperatures, heavily doped samples display lower *κ*_L_ values due to the suppression of bipolar thermal conductivity by high carrier concentration (Figure 1e). The combination of an enhanced power factor and suppressed *κ*_L_ yields a peak *ZT* of ~1.20 at 723 K for PbTe_0.996_I_0.004_ (Figure 1f). The corresponding room-temperature carrier concentration is ~1.83 × 10^19^ cm^−3^ (Figure S3a), which aligns well with the reported optimum range for n-type PbTe at 300 K [29,30]. Building upon this optimized baseline, Ga is introduced as a deep-level donor to dynamically regulate the carrier concentration, aiming to further elevate the thermoelectric performance across the entire operating temperature range.

**Figure 2:**
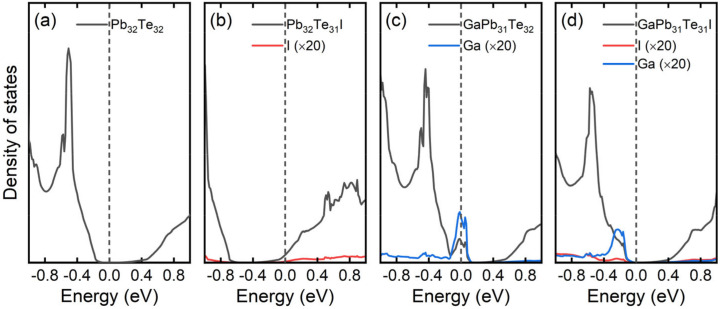
First-principles calculations of the density of states (DOS): (**a**) pristine PbTe; (**b**) I-doped PbTe; (**c**) Ga-doped PbTe; and (**d**) Ga/I co-doped PbTe. The Fermi level (dashed line) is set to zero. The partial DOS of iodine (red solid line) and gallium (blue solid line) atoms is multiplied by 20 to enhance the visibility of the dopant-induced electronic states.

To elucidate the roles of deep and shallow level impurity states in the Ga/I co-doped PbTe system, first-principles calculations were performed for pristine PbTe, I-doped PbTe, Ga-doped PbTe, and Ga/I co-doped PbTe. Since Ga may either substitute for Pb or occupy interstitial sites, various defect configurations were constructed (Figure S4) and their defect formation energies were evaluated. As detailed in Table S2, the substitutional configuration exhibits a significantly lower formation energy (0.909 eV) compared to the interstitial one (1.583 eV), indicating a strong thermodynamic preference for Ga to occupy the Pb lattice site. Based on this result, the density of states (DOS) for Pb_32−*y*_Te_32−*x*_Ga*_y_*I*_x_* (*x* = 0, 1; *y* = 0, 1) was calculated using the substitutional model (Figure 2). Iodine doping shifts the Fermi level into the conduction band, corroborating its function as a shallow donor. Conversely, Ga doping introduces a localized impurity state near the Fermi level, providing qualitative support for the formation of Ga-related deep-level defect states. In the Ga/I co-doped system, this impurity state shifts below the Fermi level, implying an electronic interaction between the I-dopants and the Ga-induced states. It should be noted that these neutral-defect calculations are intended to provide a qualitative picture of dopant-induced electronic states rather than to quantitatively establish the thermal activation energy or charge-transition levels. Nevertheless, these theoretical insights corroborate the presence of Ga-induced impurity states, which likely contribute to the complex thermally activated carrier behavior observed experimentally at elevated temperatures.

**Figure 3:**
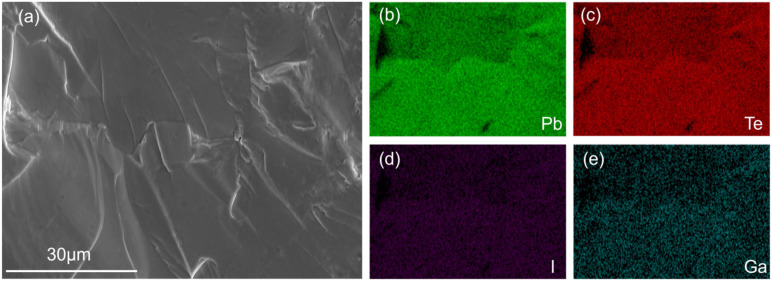
Microstructural characterization of the optimal Ga_0.02_Pb_0.98_Te_0.996_I_0.004_ sample: (**a**) SEM micrograph of the fractured surface; (**b**–**e**) corresponding EDS maps illustrating the spatial distribution of Pb, Te, I, and Ga, respectively.

The XRD patterns for the co-doped series Ga*_y_*Pb_1−*y*_Te_0.996_I_0.004_ (*y* = 0.01, 0.02, 0.03, 0.04) are shown in Figure S5a. All diffraction peaks can be perfectly indexed to the face-centered cubic PbTe phase of $Fm\bar{3}m$ symmetry, with no detectable secondary phases. A systematic shift of the diffraction peaks toward higher angles with increasing Ga content reveals a contraction of the lattice parameter (Figure S5b), which is consistent with the successful substitution of Ga on the Pb sublattice, given the smaller atomic radius of Ga relative to Pb. Furthermore, scanning electron microscopy (SEM) observation of the fractured surface of Ga_0.02_Pb_0.98_Te_0.996_I_0.004_ reveals a highly dense microstructure. Corresponding energy-dispersive X-ray spectroscopy (EDS) mapping confirms a homogeneous distribution of Pb, Te, I, and Ga, with no discernible elemental segregation (Figure 3).

**Figure 4:**
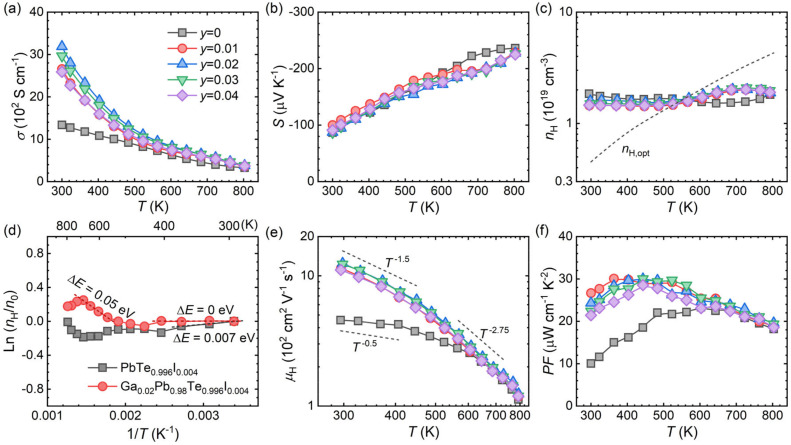
Temperature-dependent electric transport properties of Ga*_y_*Pb_1-*y*_Te_0.996_I_0.004_ (*y* = 0, 0.01, 0.02, 0.03, 0.04) bulk samples: (**a**) electrical conductivity; (**b**) Seebeck coefficient; (**c**) Hall carrier concentration; (**d**) Arrhenius plot of ln(*n*_H_/*n*_0_) versus 1/*T* for the PbTe_0.996_I_0.004_ baseline and the optimal Ga_0.02_Pb_0.98_Te_0.996_I_0.004_ composition, illustrating the estimated activation energy offset; (**e**) Hall mobility; (**f**) power factor.

Figure 4 summarizes the temperature-dependent electrical transport properties of Ga*_y_*Pb_1−*y*_Te_0.996_I_0.004_ samples. At room temperature, *σ* initially increases with Ga content, peaking at *y* = 0.02, before declining at higher Ga concentrations (Figure 4a). All samples exhibit a monotonic decrease in *σ* with rising temperature, which is characteristic of degenerate semiconductor behavior. As shown in Figure 4b, the Seebeck coefficient is nearly independent of the Ga content at lower temperatures; however, it decreases noticeably in the Ga/I co-doped samples at elevated temperatures. This deviation is attributed to the additional electron donation from thermally ionized Ga-related deep levels at high temperature. Accordingly, the Hall carrier concentration (*n*_H_) in Figure 4c exhibits only a weak temperature dependence at low temperature but begins to rise significantly at intermediate to high temperatures (above ~563 K), gradually approaching the optimal carrier concentration (*n*_H,opt_). This behavior indicates the thermal ionization of Ga-induced deep impurity levels at elevated temperatures, a phenomenon consistent with previous studies [32,33]. Furthermore, it should be noted that this apparent increase in the measured Hall carrier concentration may also be influenced by the inherent conduction band nonparabolicity of n-type PbTe, the temperature-dependent effective mass, and the resulting variations in the Hall factor at elevated temperatures [29,44]. Although the carrier concentration may not be perfectly optimal, Ga incorporation ensures that the carrier concentration remains closer to the optimal range over a much broader temperature interval compared to the singly I-doped samples.

To further analyze the temperature-dependent carrier dynamics, the relationship between ln(*n*_H_) and 1/*T* for the PbTe_0.996_I_0.004_ and Ga_0.02_Pb_0.98_Te_0.996_I_0.004_ samples was analyzed using the Arrhenius expression *n*(*T*) = *n*_0_exp(−∆*E/k*_B_*T*) [45], where ∆*E* is the apparent activation energy, and *n*_0_ is the Hall carrier concentration at 300 K. As shown in Figure 4d, within the temperature range of 563–683 K, the Ga_0.02_Pb_0.98_Te_0.996_I_0.004_ sample exhibits an activation energy of ∆*E* ≈ 0.05 eV. Rather than serving as a direct measurement of the Ga deep-level position, this value should be interpreted as an apparent activation energy. It reflects a synergistic combination of thermally activated carrier excitation from Ga-induced deep defect states, with possible contributions from the inherent conduction band nonparabolicity of n-type PbTe and temperature-dependent variations in the Hall factor at elevated temperatures [29,44]. In contrast, the baseline PbTe_0.996_I_0.004_ sample exhibits a much smaller activation energy of 0.007 eV in low temperature regime, corresponding to the shallow donor level of iodine (Figure S6b). Interestingly, at low temperatures, Ga_0.02_Pb_0.98_Te_0.996_I_0.004_ displays a nearly temperature-independent ∆*E*~0 eV (Figure S6c), indicating that the conduction mechanism in this temperature regime does not strictly adhere to a simple Arrhenius thermal activation process.

As shown in Figure 4e, the Hall mobility *μ*_H_ of all samples decreases monotonically with increasing temperature, indicating progressively strengthened carrier scattering. For the PbTe_0.996_I_0.004_ baseline, the relatively low *μ*_H_ and its weak temperature dependence (*μ*_H_~*T*^−0.5^) demonstrate that charge transport is predominantly limited by defect and/or ionized-impurity scattering. Strikingly, upon Ga incorporation, the low-temperature *μ*_H_ is markedly enhanced to over 1000 cm^2^·V^−1^·s^−1^, exhibiting a temperature dependence of *μ*_H_~*T*^−1.5^. This shift signifies that defect-dominated scattering is effectively suppressed, returning the system to a more intrinsic, acoustic-phonon-limited transport regime. This behavior contrasts sharply with previously reported Ga/I co-doped PbTe system, where heavy alloy scattering severely degraded the room-temperature Hall mobility to below 150 cm^2^·V^−1^·s^−1^ [36]. The exceptional mobility preserved in our work stems from synergistically combined factors. Primarily, the low ionization degree of Ga-related deep levels at low temperature significantly reduces ionized-impurity scattering compared to conventional heavily doped shallow donors. Additionally, inherent differences in carrier concentration, coupled with a highly optimized synthesis route that ensure better crystallinity and compositional homogeneity, effectively minimize defect-induced scattering. This interpretation is consistent with previous report on Ga-doped PbTe [32]. At elevated temperatures, the mobility decreases more steeply (*μ*_H_~*T*^−2.75^), reflecting additional scattering contributions presumably from a temperature-dependent effective mass [46], band nonparabolicity, and/or optical-phonon scattering [47,48].

**Figure 5:**
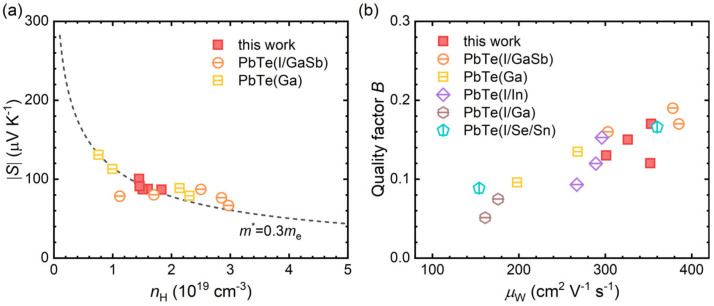
Evaluation of room-temperature thermoelectric performance indicators. (**a**) Room-temperature Seebeck coefficient versus Hall carrier concentration (Pisarenko plot). (**b**) Calculated material quality factor (*B*) as a function of weighted mobility (*μ*_W_) for the Ga*_y_*Pb_1−*y*_Te_0.996_I_0.004_ (*y* = 0.01, 0.02, 0.03, 0.04) series, with relevant literature data included for comparison [33,34,36,49,50].

Driven by this simultaneous carrier optimization and mobility enhancement, the power factor is markedly improved in the low-to-intermediate temperature range, culminating in a peak power factor of 30 μW·cm^−1^·K^−2^ for Ga_0.02_Pb_0.98_Te_0.996_I_0.004_ (Figure 4f). Consequently, the average power factor across 300–803 K is significantly elevated, and it was comparable with other n-type PbTe-based samples reported previously (Figure S7) [33,34,36,49,50]. A quantitative analysis of the electrical transport properties was conducted using the single parabolic band (SPB) model. Figure 5a shows the room-temperature Pisarenko plot (Seebeck coefficient versus carrier concentration) for the Ga*_y_*Pb_1−*y*_Te_0.996_I_0.004_ series. The experimental data are well-described by an effective mass of m* = 0.30 *m*_e_, which is highly consistent with previously reported values for Ga-doped PbTe [33,50]. This implies that Ga/I co-doping induces negligible changes to m*.

**Figure 6:**
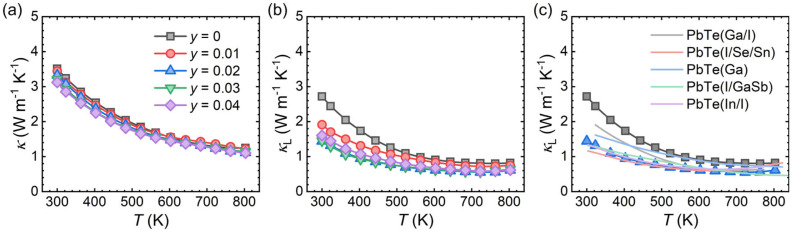
Temperature-dependent thermal transport properties of Ga*_y_*Pb_1-*y*_Te_0.996_I_0.004_ (*y* = 0, 0.01, 0.02, 0.03, 0.04) bulk samples: (**a**) Total thermal conductivity; (**b**) lattice thermal conductivity; (**c**) comparison of the lattice thermal conductivity with state-of-the-art n-type PbTe-based composition from the literature [33,34,36,49,50].

The thermal transport properties of the Ga*_y_*Pb_1−*y*_Te_0.996_I_0.004_ series are presented in Figure 6. For all compositions, the total thermal conductivity, calculated from the specific heat capacity, the measured thermal diffusivity and mass density (Figure S8a,b), generally decreases with both increasing Ga content and rising temperature. The electronic thermal conductivity (*κ*_e_) was estimated using the Wiedemann–Franz law (*κ*_e_ = *LσT*); the temperature-dependent *L* and *κ*_e_ are shown in Figure S8c,d. As shown in Figure 6b, the lattice thermal conductivity (*κ*_L_ = *κ* − *κ*_e_) is significantly reduced by Ga incorporation. At room temperature, *κ*_L_ decreases from 2.72 W·m^−1^·K^−1^ for the baseline PbTe_0.996_I_0.004_ to 1.44 W·m^−1^·K^−1^ for Ga_0.02_Pb_0.98_Te_0.996_I_0.004_, representing a substantial ~47% reduction. Because all samples exhibit uniformly high relative densities (Figure S8a) and were consolidated under identical sintering conditions, variations in porosity, grain size, and microstructural boundary scattering are considered negligible across the series. Consequently, the reduction in *κ*_L_ is mainly attributed to enhanced phonon scattering by point defects. As the temperature increases, *κ*_L_ continues to decline, reaching a minimum of ~0.55 W·m^−1^·K^−1^ at 723 K for the Ga_0.02_Pb_0.98_Te_0.996_I_0.004_ sample. Figure 6c provides a comprehensive comparison, clearly illustrating that the *κ*_L_ achieved in this work ranks among the lowest values reported for n-type PbTe materials [33,34,36,49,50].

Having established both the electrical transport characteristics and the significant reduction in lattice thermal conductivity, we evaluated the overall material quality factor (*B*), which serves as a comprehensive metric for thermoelectric performance potential [51,52]. As shown in Figure 5b, *B* is plotted as a function of *μ*_W_ and compared alongside representative n-type PbTe systems from the literature [33,34,36,49,50]. Notably, the data from this work lies distinctly above those of previously reported PbTe(I/Ga) systems [36]. This demonstrates that our optimized synthesis route, coupled with more precise carrier concentration tuning, leads to a substantially enhanced weighted mobility and a correspondingly higher quality factor. Overall, the fundamental transport merit achieved here remains highly competitive among state-of-the-art n-type PbTe materials [33,34,36,49,50].

**Figure 7:**
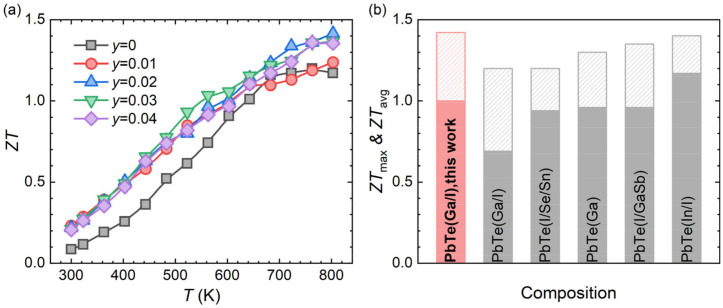
Thermoelectric figure of merit for the Ga*_y_*Pb_1-*y*_Te_0.996_I_0.004_ (*y* = 0, 0.01, 0.02, 0.03, 0.04) series: (**a**) temperature-dependent *ZT*; (**b**) comparison of the maximum *ZT* (*ZT*_max_) and average *ZT* (*ZT*_avg_) values with state-of-the-art n-type PbTe-based materials from the studies [33,34,36,49,50].

The temperature-dependent dimensionless figure of merit is presented in Figure 7a. Benefiting from the synergistically enhanced power factor and suppressed *κ*_L_, all Ga/I co-doped samples exhibit superior *ZT* values across the entire measured temperature range compared to the singly doped PbTe_0.996_I_0.004_ baseline. To ensure an equitable comparison with existing literature, the average *ZT* (*ZT*_avg_) was calculated by integrating *ZT* over the temperature range of 400–773 K, accommodating the measurement limitations present in several prior reports. Among the investigated compositions, the Ga_0.02_Pb_0.98_Te_0.996_I_0.004_ sample achieves a maximum *ZT* of ~1.41 at 803 K and a *ZT*_avg_ of ~1.00 within 400–773 K range. This represents a substantial ~25% improvement in average performance over the baseline PbTe_0.996_I_0.004_ (*ZT*_avg_ ~ 0.80). A benchmark comparison with state-of-the-art n-type PbTe materials (Figure 7b) confirms that the thermoelectric performance achieved here is highly competitive [33,34,36,49,50]. Collectively, these findings strongly validate the success of the deep-shallow-level engineering strategy utilizing Ga and I co-doping. This work not only demonstrates an effective means to decouple and optimize the electrical and thermal transport properties, but also enriches the theoretical understanding of deep-shallow co-doping, providing a robust framework for the systematic advancement of n-type PbTe and analogous lead chalcogenides.

## 4. Conclusions

In summary, high-performance n-type Ga/I co-doped PbTe bulk materials were successfully synthesized via a melting and annealing process followed by spark plasma sintering. We demonstrate that iodine acts as an effective shallow donor to establish an optimal baseline electron concentration, while the incorporation of gallium introduces deep-level defect states. The thermal ionization of these Ga-induced states enables a dynamic, temperature-adaptive tuning of the carrier concentration. Concurrently, Ga doping enhancing carrier mobility. This synergistic carrier regulation and mobility enhancement sustains an elevated power factor across a broad temperature range. Moreover, the introduction of Ga significantly intensifies point-defect phonon scattering, which markedly suppresses the lattice thermal conductivity. Through this simultaneous optimization of electrical and thermal transport properties, the optimal composition, Ga_0.02_Pb_0.98_Te_0.996_I_0.004_, achieves a peak *ZT* of 1.41 at 803 K and a competitive *ZT*_avg_ of 1.00 within 400–773 K. Ultimately, the successful implementation of this temperature-adaptive design strategy proves highly effective for sustaining superior thermoelectric performance across wide thermal gradients, accelerating the development of high-efficiency of n-type PbTe thermoelectrics.

## Data Availability

The original contributions presented in this study are included in the article/Supplementary Material. Further inquiries can be directed to the corresponding authors.

## Supplementary Materials

The following supporting information can be downloaded at: https://www.mdpi.com/article/10.3390/ma19132832/s1, Figure S1: The calculated temperature-dependent heat capacity of Ga*_y_*Pb_1-*y*_Te_0.996_I_0.004_ (*y* = 0, 0.01, 0.02, 0.03, 0.04). Figure S2: X-ray diffraction patterns for PbTe_1-*x*_I*_x_* (*x* = 0.002, 0.003, 0.004, 0.005) samples. Figure S3: Temperature-dependent electrical transport properties of PbTe_1-*x*_I*_x_* (*x* = 0.002, 0.003, 0.004, 0.005): (a) Carrier concentration; (b) Carrier mobility. Figure S4: The structures of the supercell used in the calculation. (a) substitution (sub); and (b) interstitial (int). Figure S5: Structure characterization of Ga*_y_*Pb_1-*y*_Te_0.996_I_0.004_ (*y* = 0, 0.01, 0.02, 0.03, 0.04). (a) XRD patterns with magnified view of peak at 2θ ~ 27.6° (right panel). Vertical tags at the bottom indicate the Bragg reflections of PbTe (JCPDS 65-0159). (b) Refined lattice parameters as a function of Ga content. Figure S6: Band structure: (a) I-doped PbTe; (b) Enlarged detail of I-doped PbTe along R-Γ-X path around Γ point; and (c) Ga/I co-doped PbTe. Figure S7: Comparison of average power factor with reported n-type PbTe-based samples. Figure S8: Thermal transport properties of Ga*_y_*Pb_1-*y*_Te_0.996_I_0.004_ (*y* = 0, 0.01, 0.02, 0.03, 0.04) (a) Measured density (*D*_m_), Theoretical density (*D*_t_) and numbers are relative density; (b) Thermal diffusivity; (c) Lorenz number; (d) Electronic thermal conductivity; Table S1: The density and relative density of Ga*_y_*Pb_1-*y*_Te_0.996_I_0.004_ samples. Table S2: Comparison of defect formation energy of sub and int models. Table S3: Elemental compositions of Ga/I co-doped PbTe samples determined by micro-area EDS analysis (Average of 15 micro-areas). Table S4: A benchmark comparison with state-of-the-art n-type PbTe materials. The *ZT*_avg_ values were calculated over the temperature range of 400–773 K, accommodating the measurement limitations present in previously reported works. References [53,54,55,56,57] are cited in the Supplementary Materials.
