# Supplementary material for: Temperature-Adaptive Carrier Regulation and Enhanced Thermoelectric Performance in n-Type PbTe via Deep-Shallow Co-Doping
Source: Materials (Basel). 2026 Jul 2;19(13):2832. doi: 10.3390/ma19132832 (PMC13363260; doi:10.3390/ma19132832)
Supplement: Supplementary file 1 [file materials-19-02832-s001.zip › materials-4393941-supplementary.pdf]

Temperature-Adaptive Carrier Regulation and Enhanced Thermoelectric Performance in n-Type PbTe via Deep-Shallow Co-Doping

Aihua Song [1], Peng Zhao [1,*], Binhao Wang [2], Dan Wang [1], Chen Chen [1], Tao Shen [1], Hang Li [1], Bo Xu [1,*] and Yongjun Tian [1]

[1] Center for High Pressure Science (CHiPS), State Key Laboratory of Metastable Materials Science and Technology, Yanshan University, Qinhuangdao 066004, China

[2] State Key Laboratory for High Performance Tools, Zhengzhou Abrasive Grinding Research Institute Co., Ltd., Zhengzhou 450001, China

* Correspondence: zhaopeng@ysu.edu.cn (P.Z.); bxu@ysu.edu.cn (B.X.)

1.1 Details for the single parabolic band model

In single parabolic band (SPB) model with the assumption of acoustic phonon scattering approximation ($r = -1/2$), the Seebeck coefficient and carrier concentration can be expressed as [1, 2]:

$$S = -\frac{k_{\mathrm{B}}}{e}\left(\frac{2F_1(\eta)}{F_0(\eta)} - \eta\right) \tag{S1}$$

$$n_{\mathrm{H}} = 4\pi\left(\frac{2m^* k_{\mathrm{B}} T}{h^2}\right)^{3/2} F_{1/2}(\eta) \tag{S2}$$

where η is the reduced Fermi energy, k_{B} and h are the Boltzmann constant and Planck constant, respectively, and $F_j(\eta) = \int_0^\infty \frac{\varepsilon^j d\varepsilon}{1+e^{\varepsilon-\eta}}$ is the j-th Fermi integral with ε being the reduced energy.

The carrier mobility is expressed as:

$$\mu_{\mathrm{H}} = \frac{\sqrt{2}\pi e\hbar^4}{3(k_{\mathrm{B}}T)^{3/2}} \frac{v_{\mathrm{L}}^2 D}{\Xi^2 m_{\mathrm{b}}^{5/2}} \frac{F_0(\eta)}{F_{1/2}(\eta)} \tag{S3}$$

where $\hbar$ is the reduced Planck constant, v_{L} is the sound velocity, D is the sample density, Ξ is the deformation potential, m_{b} is the band effective mass, and its relationship with m^* is $m^* = N^{2/3}\, m_{\mathrm{b}}$, where N is the band degeneracy. The electrical resistivity can be expressed as:

$$\frac{1}{\rho} = n_{\mathrm{H}} e \mu_{\mathrm{H}} \tag{S4}$$

and then the power factor will be

$$PF = \frac{S^2}{\rho} \tag{S5}$$

In addition, the $n_{\mathrm{H,opt}}$ curve was calculated based on the SPB model by maximizing the power factor as a function of carrier concentration at each temperature [3]. The calculated $n_{\mathrm{H,opt}}$ should be regarded as a reference carrier-concentration window for achieving favorable electrical transport, rather than an exact universal value.

To evaluate the electrical transport performance of thermoelectric materials, the weighted mobility (μ_{W}) is introduced [4], which describes the relationship between the Seebeck

coefficient (S) and electrical conductivity (σ). The μ_W value of a material can be calculated from the measured S and σ values according to the following equation[5]:

$$\sigma = \frac{8\pi e}{3}\left(\frac{2m_e k_B T}{h^2}\right)^{3/2} F_0(\eta)\mu_W \qquad \text{(S6)}$$

and quality factor (B) can be expressed as [5, 6]:

$$B = \frac{k_B^2}{e^2}\frac{8\pi e(2m_e k_B)^{3/2}}{3h^3}\frac{\mu_W T^{5/2}}{\kappa_L} \qquad \text{(S7)}$$

1.2 Defect formation energy

The defect formation energy of structural models is calculated as a function of chemical potentials and can be written as [7, 8]:

$$E^{form} = E_{total}^{defect} - E_{total}^{perfect} + \sum n_i \mu_i \quad \text{(S8)}$$

$$\mu_i = E_i / N_i \quad \text{(S9)}$$

Where E^{form} is the defect formation energy, E_{total}^{defect} is the total energy of the supercell with the defect, $E_{total}^{perfect}$ is the total energy of the PbTe supercell, μ_i is the chemical potential of element Ga and Pb. n_i is the number of Ga and Te atoms that were added ($n_i < 0$) or removed ($n_i > 0$) to form the system. E_i is the total energy of the solid structure of Ga and Pb. N_i is the total number of atoms in a solid structure of Ga and Pb.

1.3 The calculation of average *ZT*

The average *ZT* (ZT_{avg}) was calculated according to the following formula:

$$ZT_{avg} = \frac{1}{T_h - T_c}\int_{T_c}^{T_h} ZTdT \quad \text{(S10)}$$

where T_c and T_h are the lower and upper temperatures of the investigated range, respectively. To ensure a rigorous and equitable comparison across different studies, all ZT_{avg} values were standardized over the 400–773 K temperature range, accommodating the measurement limitations present in several previously reported works.

Table S1. The density and relative density of $Ga_yPb_{1-y}Te_{0.996}I_{0.004}$ samples.

Sample	Measured density (g cm^{-3})	Theoretical density (g cm^{-3})	Relative density
y=0	8.070	8.222	98%
y=0.01	7.989	8.203	97%
y=0.02	7.954	8.169	97%
y=0.03	7.928	8.139	97%
y=0.04	7.873	8.109	97%

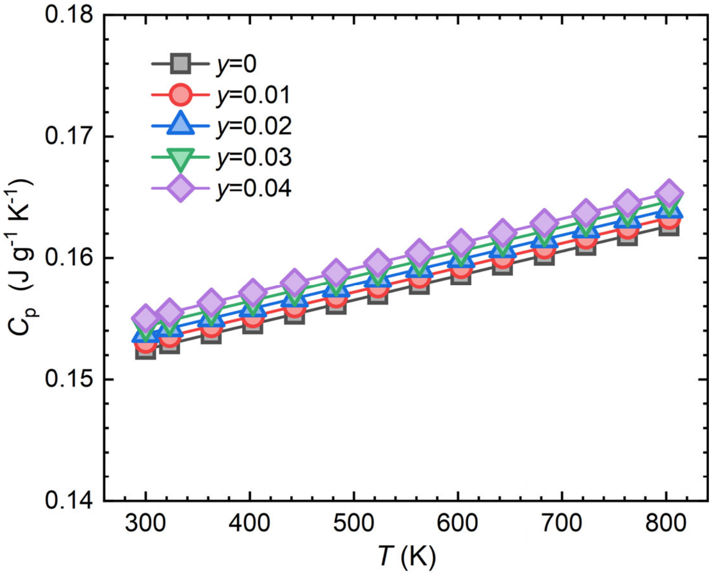

Figure S1. The calculated temperature-dependent heat capacity of $Ga_yPb_{1-y}Te_{0.996}I_{0.004}$ (y = 0, 0.01, 0.02, 0.03, 0.04).

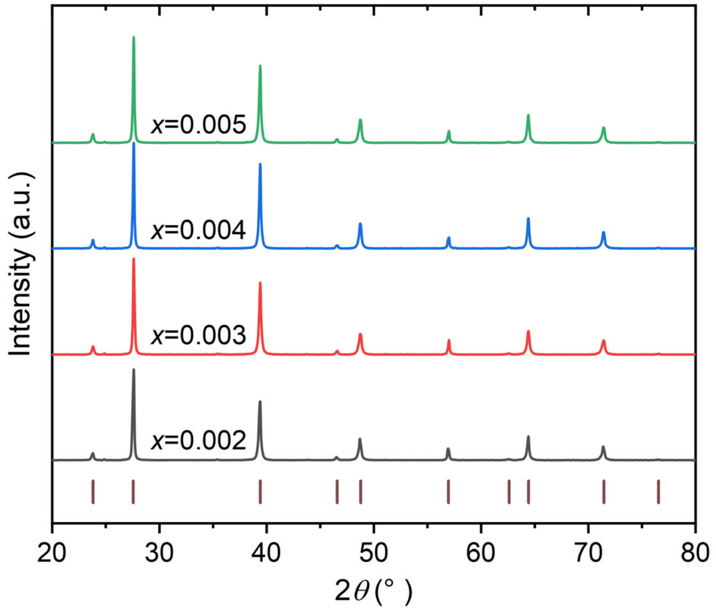

Figure S2. X-ray diffraction patterns for $PbTe_{1-x}I_x$ (x = 0.002, 0.003, 0.004, 0.005) samples.

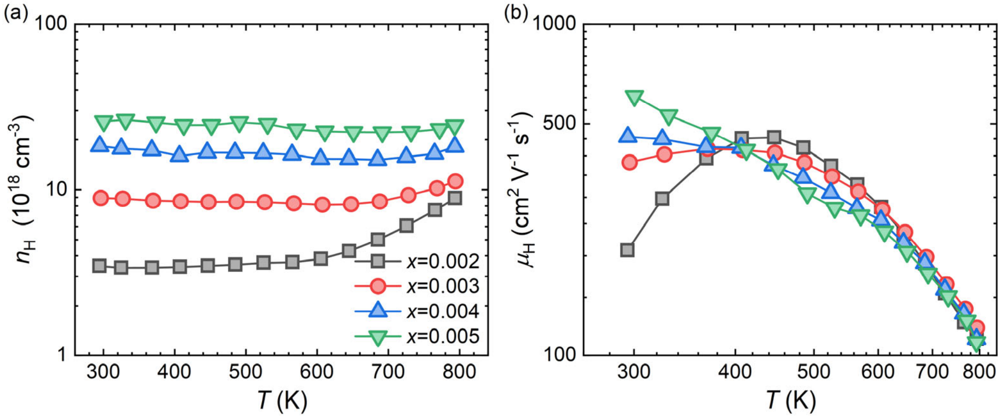

Figure S3. Temperature-dependent electrical transport properties of $PbTe_{1-x}I_x$ ($x = 0.002$, 0.003, 0.004, 0.005): (**a**) Carrier concentration; (**b**) Carrier mobility.

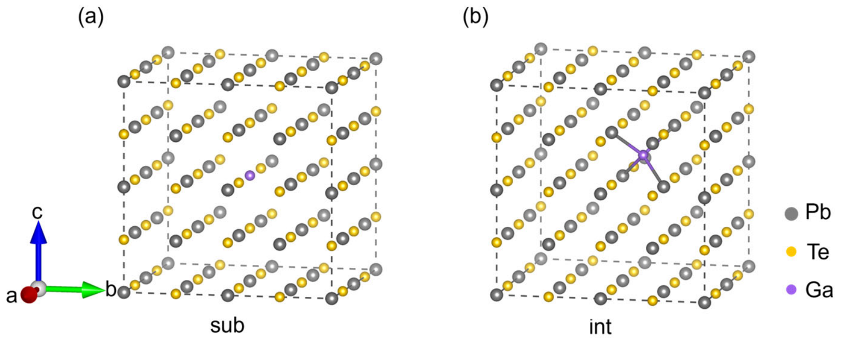

Figure S4. The structures of the supercell used in the calculation. (**a**) substitution (sub); and (**b**) interstitial (int).

Table S2. Comparison of defect formation energy of sub and int models.

Models	Defect formation energy (eV)
sub	0.909
int	1.583

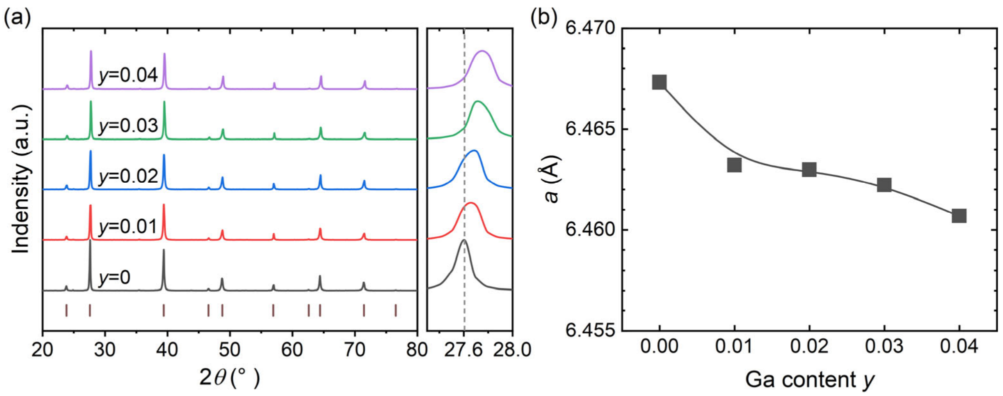

Figure S5. Structure characterization of $Ga_yPb_{1-y}Te_{0.996}I_{0.004}$ (y = 0, 0.01, 0.02, 0.03, 0.04). (**a**) XRD patterns with magnified view of peak at $2\theta \sim 27.6^{\circ}$ (right panel). Vertical tags at the bottom indicate the Bragg reflections of PbTe (JCPDS 65-0159). (**b**) Refined lattice parameters as a function of Ga content.

Table S3. Elemental compositions of Ga/I co-doped PbTe samples determined by micro-area EDS analysis (Average of 15 micro-areas).

Nominal composition	Measured composition
$PbTe_{0.996}I_{0.004}$	$Pb_{0.941\pm0.011}Te_{0.996\pm0.013}I_{0}$
$Ga_{0.01}Pb_{0.99}Te_{0.996}I_{0.004}$	$Ga_{0.006\pm0.003}Pb_{0.932\pm0.011}Te_{0.996\pm0.012}I_{0}$
$Ga_{0.02}Pb_{0.98}Te_{0.996}I_{0.004}$	$Ga_{0.008\pm0.003}Pb_{0.930\pm0.012}Te_{0.996\pm0.013}I_{0}$
$Ga_{0.03}Pb_{0.97}Te_{0.996}I_{0.004}$	$Ga_{0.012\pm0.003}Pb_{0.930\pm0.011}Te_{0.996\pm0.011}I_{0}$
$Ga_{0.04}Pb_{0.96}Te_{0.996}I_{0.004}$	$Ga_{0.027\pm0.004}Pb_{0.925\pm0.013}Te_{0.996\pm0.013}I_{0}$

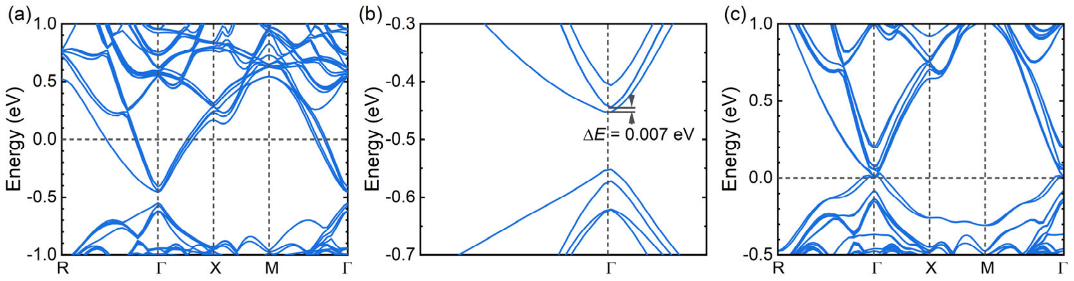

Figure S6. Band structure: (**a**) I-doped PbTe; (**b**) Enlarged detail of I-doped PbTe along R−Γ−X path around Γ point; and (**c**) Ga/I co-doped PbTe.

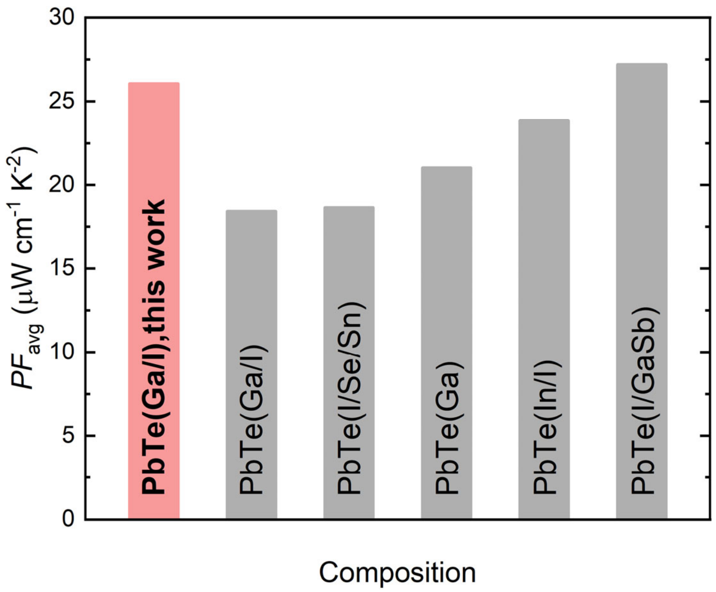

Figure S7. Comparison of average power factor with reported n-type PbTe-based samples [3, 9-12].

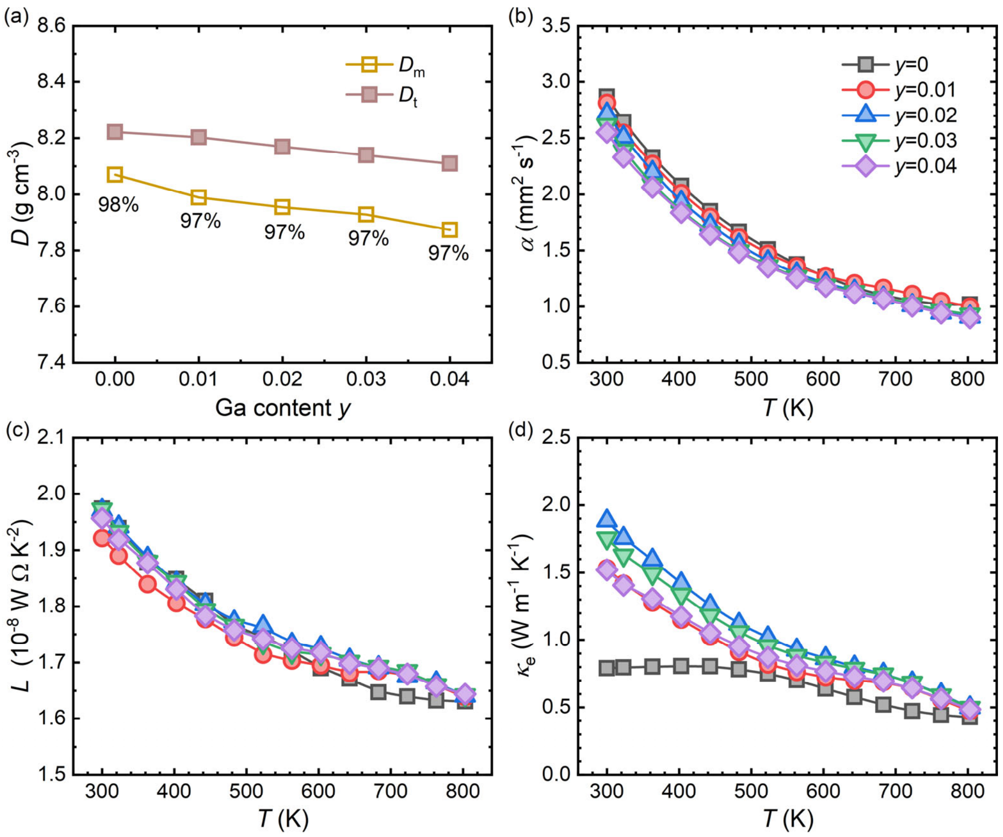

Figure S8. Thermal transport properties of $Ga_yPb_{1-y}Te_{0.996}I_{0.004}$ (y = 0, 0.01, 0.02, 0.03, 0.04) (**a**) Measured density (D_m), Theoretical density (D_t) and numbers are relative density; (**b**) Thermal diffusivity; (**c**) Lorenz number; (**d**) Electronic thermal conductivity.

Table S4. A benchmark comparison with state-of-the-art n-type PbTe materials. The ZT_{avg} values were calculated over the temperature range of 400–773 K, accommodating the measurement limitations present in previously reported works.

Material system	ZT_{max}	ZT_{avg}	Ref.
**PbTe(Ga/I)**	**1.41**	**1.00**	**This work**
PbTe(Ga/I)	1.20	0.69	Ref.[9]
PbTe(I/Se/Sn)	1.20	0.94	Ref.[10]
PbTe(Ga)	1.30	0.96	Ref.[11]
PbTe(I/GaSb)	1.35	0.96	Ref.[12]
PbTe(In/I)	1.40	1.17	Ref.[3]

References

1. Zhao, P.; Yao, H.; Zhi, S.; Ma, X.; Wu, Z.; Liu, Y.; Wang, X.; Yin, L.; Zhang, Z.; Hou, S.; Wang, X.; Chen, S.; Chen, C.; Lin, X.; Liu, H.; Liu, X.; Cao, F.; Zhang, Q.; Mao, J., Realizing n-type CdSb with promising thermoelectric performance. *J. Mater. Sci. Technol.* **2023,** 144, 54-61. https://doi.org/10.1016/j.jmst.2022.10.017.
2. Zhu, J.; Zhang, X.; Guo, M.; Li, J.; Hu, J.; Cai, S.; Cai, W.; Zhang, Y.; Sui, J., Restructured single parabolic band model for quick analysis in thermoelectricity. *npj Comput. Mater.* **2021,** 7, (1), 116. https://doi.org/10.1038/s41524-021-00587-5.
3. Zhang, Q.; Song, Q.; Wang, X.; Sun, J.; Zhu, Q.; Dahal, K.; Lin, X.; Cao, F.; Zhou, J.; Chen, S.; Chen, G.; Mao, J.; Ren, Z., Deep defect level engineering: a strategy of optimizing the carrier concentration for high thermoelectric performance. *Energy Environ. Sci.* **2018,** 11, (4), 933-940. https://doi.org/10.1039/C8EE00112J.
4. Snyder, G. J.; Snyder, A. H.; Wood, M.; Gurunathan, R.; Snyder, B. H.; Niu, C., Weighted mobility. *Adv. Mater.* **2020,** 32, (25), 2001537. https://doi.org/10.1002/adma.202001537.
5. Zhao, P.; Yu, F.; Wang, B.; Zhao, H.; Chen, C.; Wang, D.; Ying, P.; Wu, Y.; Li, P.; Zhang, B.; Liu, B.; Zhao, Z.; Hu, W.; Yu, D.; He, J.; Liu, Z.; Xu, B.; Tian, Y., Porous bismuth antimony telluride alloys with excellent thermoelectric and mechanical properties. *J. Mater. Chem. A* **2021,** 9, (8), 4990-4999. https://doi.org/10.1039/D0TA09795K.
6. He, Z.; Zhu, J.; Su, W.; An, X.; Zhao, C.; Yuan, W.; Lin, L.; Ang, R., Achieving high quality factor and enhanced thermoelectric performance in polycrystalline SnS by Ag doping and Se alloying. *Appl. Phys. Lett.* **2023,** 123, (24), 242104. https://doi.org/10.1063/5.0180362.
7. Zong, J.; Liang, Y.; Liu, F.; Zhang, M.; Song, K.; Feng, J.; Xi, B.; Xiong, S., Engineering twin structures and substitutional dopants in $ZnSe_{0.7}Te_{0.3}$ anode material for enhanced sodium storage performance. *Nat. Commun.* **2025,** 16, (1), 4406. https://doi.org/10.1038/s41467-025-59707-0.
8. Van de Walle, C. G.; Neugebauer, J., First-principles calculations for defects and impurities: Applications to III-nitrides. *J. Appl. Phys.* **2004,** 95, (8), 3851-3879. https://doi.org/10.1063/1.1682673.
9. Zong, Y.; Wang, Y.; Zhai, J.; Su, W.; Tan, C.; Li, Z.; Sun, Y.; Wang, H.; Wang, C.; Wang, H., Enhanced solubility of Ga and deep defect level lead to high thermoelectric performance of n-type $Pb_{1-x}Ga_xTe_{0.995}I_{0.005}$ alloys. *J. Phys. Chem. C* **2024,** 128, (11), 4424-4430. https://doi.org/10.1021/acs.jpcc.4c00220.
10. Xiao, Y.; Li, W.; Chang, C.; Chen, Y.; Huang, L.; He, J.; Zhao, L.-D., Synergistically optimizing thermoelectric transport properties of n-type PbTe via Se and Sn co-alloying. *J. Alloys Compd.* **2017,** 724, 208-221. https://doi.org/10.1016/j.jallcom.2017.06.296.
11. Wang, Z.; Wang, G.; Wang, R.; Zhou, X.; Chen, Z.; Yin, C.; Tang, M.; Hu, Q.; Tang, J.; Ang, R., Ga-doping-induced carrier tuning and multiphase engineering in n-type PbTe with enhanced thermoelectric performance. *ACS Appl. Mater. Interfaces* **2018,** 10, (26), 22401-22407. https://doi.org/10.1021/acsami.8b05117.
12. Sarkar, S.; Hua, X.; Hao, S.; Zhang, X.; Bailey, T. P.; Slade, T. J.; Yasaei, P.; Korkosz, R. J.; Tan, G.; Uher, C.; Dravid, V. P.; Wolverton, C.; Kanatzidis, M. G., Dissociation of GaSb in n-type PbTe: off-centered gallium atom and weak electron-phonon coupling provide high thermoelectric performance. *Chem. Mater.* **2021,** 33, (5), 1842-1851. https://doi.org/10.1021/acs.chemmater.0c04854.
